# New Insights into the Biological Role of Mammalian ADARs; the RNA Editing Proteins

**DOI:** 10.3390/biom5042338

**Published:** 2015-09-30

**Authors:** Niamh Mannion, Fabiana Arieti, Angela Gallo, Liam P. Keegan, Mary A. O’Connell

**Affiliations:** 1 Paul O’Gorman Leukaemia Research Centre, Institute of Cancer Sciences, College of Medical, Veterinary and Life Sciences, University of Glasgow, 21 Shelley Road, Glasgow G12 0ZD, UK; E-Mail: Niamh.Mannion@glasgow.ac.uk; 2 CEITEC—Central European Institute of Technology, Masaryk University, Kamenice 5, Brno 625 00, Czech Republic; E-Mails: fabiana.arieti@ceitec.muni.cz (F.A.); liam.keegan@ceitec.muni.cz (L.P.K.); 3 Oncohaematoogy Department, Ospedale Pediatrico Bambino Gesù (IRCCS) Viale di San Paolo, Roma 15-00146, Italy; E-Mail: pillipo44@yahoo.it

**Keywords:** ADAR, RNA editing, Alu elements, dsRBDs, deaminase domain, cancer

## Abstract

The ADAR proteins deaminate adenosine to inosine in double-stranded RNA which is one of the most abundant modifications present in mammalian RNA. Inosine can have a profound effect on the RNAs that are edited, not only changing the base-pairing properties, but can also result in recoding, as inosine behaves as if it were guanosine. In mammals there are three ADAR proteins and two ADAR-related proteins (ADAD) expressed. All have a very similar modular structure; however, both their expression and biological function differ significantly. Only two of the ADAR proteins have enzymatic activity. However, both ADAR and ADAD proteins possess the ability to bind double-strand RNA. Mutations in ADARs have been associated with many diseases ranging from cancer, innate immunity to neurological disorders. Here, we will discuss in detail the domain structure of mammalian ADARs, the effects of RNA editing, and the role of ADARs in human diseases.

## 1. Introduction

RNAs in the cell are rarely found without proteins attached; instead they are present as ribonucleoproteins (RNPs) complexes that are formed co-transcriptionally. RNA can also undergo a large number of modifications; over 100 have been described to date and this number is likely to be underestimated [1]. Since the nucleotide composition of a given RNA determines its chemical and structural proprieties, base modifications can either increase or decrease the stability of the RNA structures. It can also influence the repertoire of proteins binding to RNA and consequently have dramatic effects that can lead to disease. In this chapter we will focus on one of the most widespread and abundant type of post-transcriptional RNA modification/editing; enzymatic deamination of adenosine residues (A) to inosine (I) within double-stranded RNAs (dsRNAs) which is catalyzed by the ADAR (adenine deaminase acting on RNA) family of proteins.

## 2. RNA Editing

The term RNA editing was coined by Rob Benne when he discovered that the mitochondrial cytochrome oxidase subunit II gene from the trypanosomes species *Trypanosoma* *brucei* and *Crithidia* *fasciculata* contain a frameshift at amino acid 170 [2]. Four nucleotides are inserted at the frameshift position that are not encoded in the DNA and this restores the reading frame. This finding was subsequently confirmed by others who demonstrated that RNA editing was essential for the generation of functional mitochondrial proteins in trypanosomes [3]. In 1987, the field of mammalian RNA editing was established when that year the first papers were published. The initial publications were by the groups of Weintraub and Melton who discovered an activity that unwound RNA duplexes [4,5]. Initially this activity was described as an RNA helicase however it was subsequently shown to be a deaminase encoded by ADAR [6]. The second breakthrough came from the laboratory of James Scott who found that a tissue-specific RNA modification results in two distinct proteins being generated from the same primary transcript [7]. The conversion of cytosine (C) into uracil (U) by deamination generates a stop codon in the transcript encoding apolipoprotein B (apoB) is catalyzed by the apolipoprotein B mRNA-editing enzyme catalytic polypeptide 1 (APOBEC1). Thus, in the context of the time, the initial assumption was that the function of RNA editing was to restore an open reading frame or to generate alternative isoforms of the encoded protein. Therefore, it was considered to be distinct from RNA modification that modifies RNA without creating protein diversity. However, in hindsight, are RNA editing and RNA modification two sides of the same coin? 

**Figure 1:**
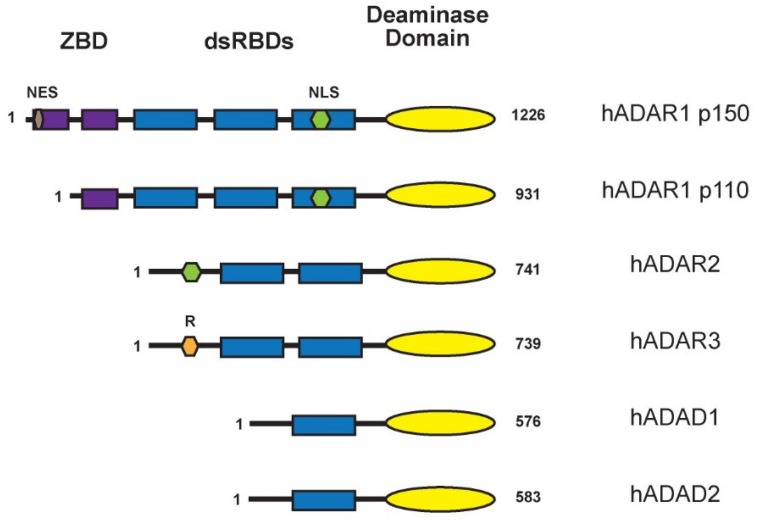
Domain organization of ADAR and ADAD proteins expressed in *Homo sapiens*. All of the proteins contain N-terminal dsRBDs and C-terminal deaminase domains. An arginine rich (R) region is present at the N-terminus of ADAR3. There are two isoforms of hADAR1, ADAR1 p110 and ADAR1p150, both of which contain a Z-DNA binding domains (ZBD) at the N-terminal.

The cytidine deaminase (CDA) super family of enzymes includes both the ADAR and the APOBEC family of proteins as well as adenosine deaminase acting on the tRNA (ADAT) family. These enzymes catalyze deamination and their catalytic domain is evolutionarily conserved [8]. Following the identification of ADAR1 [9,10,11] the search for other ADAR-like genes began and this led to the discovery of ADAR2 (also known as ADARB1) [12,13,14]. The brain-specific ADAR3 (also known as ADARB2) was also identified based on its homology with ADAR2 [15]. A screen for genes that encode RNA binding proteins led to the identification of the testis nuclear RNA binding protein (TENR) [16]. TENR is testis-specific and found to contain a catalytically-inactive deaminase domain [16]. Due to the annotation of a second testis-specific gene encoding an adenosine deaminase domain containing protein (ADAD2), TENR has been renamed to ADAD1. Thus, there are three ADAR genes and two ADAD genes which are ADAR related in mammals: ADAR1 and ADAR2 are catalytically active, whereas ADAR3, ADAD1, and ADAD2 are not enzymatically active, but are dsRNA binding proteins (Figure 1). 

## 3. Domain Organization and Structure of ADAR Enzymes

ADARs are modular proteins containing similar functional domains. All have at their N-terminus double-stranded RNA binding domains (dsRBDs), which make direct contacts with the dsRNA substrates whereas the catalytic deaminase domain is located at the C-terminus (Figure 1). Some have distinct domains not present in other family members.

The *ADAR1* gene was mapped to the q21.1–21.2 locus on chromosome one in humans [17]. It extends ~40 kb and contains 17 exons [18]. There are two different isoforms of ADAR1; a 150 kDa protein (ADAR1 p150) that is induced by interferon (IFN) and a 110 kDa protein (ADAR1 p110) that is constitutively expressed [19]. The two isoforms have distinct translation initiation sites so that ADAR1p150 starts translation in exon 1A (AUG1) whilst the ADAR1p110 protein initiates from exon two (AUG296) [19,20]. The generation of different ADAR1 isoforms is due to the alternative splicing of the exon one. An IFN-inducible promoter located in exon 1A contains a 12-bp IFN-stimulated response element (ISRE), thereby generating the transcript coding for the longer ADAR1p150 isoform [21]. However, transcripts encoding ADAR1p110 are generated from multiple promoters located within exon 1B, 1C and exon two [21,22]. As neither exon 1B nor exon 1C contain translation initiation sites, translation starts at the AUG296 codon within exon two. Additional alternative splicing events have been identified within exon 7 to generate variants that contain either exon 7a or 7b [20]. Exon 7b is missing 26 amino acids that are present in exon 7a [20]. Furthermore, exon 7b is associated with ADAR1p150 transcripts whereas exon 7a is present in the constitutively-expressed ADAR1p110 transcripts [23].

A unique feature of ADAR1 when compared to other members of the ADAR family, is the presence of Z-DNA binding domains (ZBDs) at the *N*-terminus. The Z-alpha (Zα) domain is exclusive to the ADAR1p150 isoform whereas both ADAR1p150 and ADAR1p110 contain a Zβ domain [24]. The ZBDs bind DNA/RNA that is in a left handed conformation [25]; however, only the Zα domain has Z-DNA/RNA binding capacity [26]. Although the functional significance of the ZBDs in ADAR1 is unclear, all proteins containing Zα domains have been implicated in the type I IFN response pathway [27]. Furthermore, the Zα domain is essential for the localization of ADAR1p150 to cytoplasmic stress granules following activation of type I IFN-induced stress [28].

*In vitro* analysis of recombinant ADAR3 demonstrated that it can bind both dsRNA and ssRNA [29]. Site-directed mutagenesis of ADAR3 revealed that it binds ssRNA via a unique arginine/lysine rich domain (R-domain) located in the *N*-terminus. This R-domain is important for determining the subcellular localization of ADAR3 and acts as a nuclear localization signal (NLS) [30]. The importin alpha-1 factor, KPNA2 recognizes and interacts with this R-domain to promote the nuclear import of ADAR3.

## 4. dsRNA Binding Domains of ADARs

**Figure 2:**
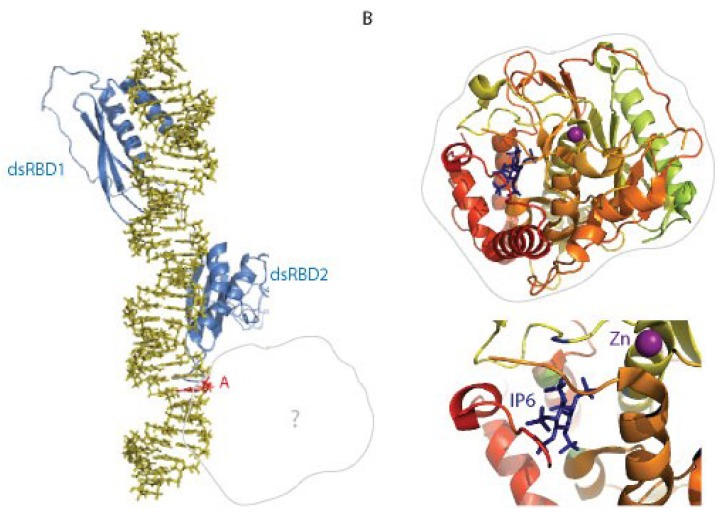
Structural information of ADAR enzymes. (**A**) NMR structure of the dsRBD1 and dsRBD2 of hADAR2 (blue) in complex with the RG stem loop RNA target [37]. The specific adenosine that is deaminated in the catalytic reaction is highlighted in red. In grey is shown where the deaminase domain is likely positioning during the reaction. PDB file: 2L3J. (**B**) Crystal structure of the catalytic domain of hADAR2 [41], secondary structures are colored from yellow (N-terminus) to red (C-terminus). The Zinc ion and inositol hexakisphosphate molecule (IP6) cofactor are highlighted and colored in blue. PDB file: 1ZY7.

All proteins that bind to dsRNA contain an evolutionarily-conserved 65-68 amino acid homologous sequence that is known as dsRBD [31]. It was originally identified in *Drosophila Staufen* and *Xenopus* Xlrbpa proteins [31]. Nuclear magnetic resonance (NMR) studies have characterized the canonical dsRBD fold with a topology of: α-helix 1, β-strand 1, β-strand 2, β strand 3, and α-helix 2 (αβββα) [32,33,34]. The two α-helices lie on one side of the three-stranded anti-parallel β-sheet creating a hydrophobic core that makes contact with the RNA substrate (Figure 2a) [35]. In particular, the N-terminal tip of the α-helix 2 plays a significant role in the interactions between the dsRBD and the dsRNA substrate. This particular region is part of the dsRBD consensus sequence and consists of a cluster of lysines, KKXXK (where X is any amino acid) [31]. *In vitro* mutagenesis studies of these lysine residues have demonstrated their importance for both the binding and editing function of ADARs [36,37,38].

NMR chemical shift perturbation studies of ADAR2 bound to a 71nt hairpin RNA encoding the R/G editing site of the *Gria2* transcript revealed that dsRBD1 recognizes a conserved pentaloop whereas the dsRBD2 binds two bulges close to the editing site (Figure 2) [36,37]. Stefl and colleagues also demonstrate that the orientation of α-helix 1 is different between the two dsRBD and this affects the interactions with α-helix 2 in the respective dsRBD. Additionally, the conformation of the β1-β2 loop also differs between the two dsRBDs [36,37]. These results suggest that dsRNA substrate recognition by ADARs is achieved through their dsRBDs via recognition of a specific structure. 

Although all dsRBDs have a consensus sequence that is essential for dsRNA binding, each protein differs functionally and this is reflected in their RNA binding specificity. Liu and colleagues investigated whether the dsRBDs contributed to the activity of ADAR1 [39]. Chimeric proteins were created whereby the dsRBDs of ADAR1 were substituted with those of PKR. Analysis of the recombinant PKR-ADAR1 chimera revealed that the editing activity was significantly impaired compared to wild-type ADAR1 [39]. Furthermore, small inhibitory RNA substrates that bind PKR were also able to inhibit the editing activity of the PKR-ADAR1 chimera. These results demonstrate that the dsRBDs play a role in substrate specificity which in turn influences the function of ADARs.

Differences also exist between the dsRBDs amongst the ADAR proteins. Mutagenic analysis of ADAR1 revealed that deletion of dsRBD2 has no effect on the editing activity of the enzyme whereas the deletion of dsRBD1 or dsRBD3 significantly reduces the its editing activity [40]. Interestingly, the two dsRBDs of ADAR2 align to dsRBD1 and dsRBD3 of ADAR1 [13]. Analysis of the two dsRBDs in ADAR2 demonstrated that dsRBD2 is required for editing of all substrates whereas dsRBD1 is only required for editing of a specific subset of substrates [38]. Furthermore, point mutations in dsRBD2 only affected editing of some of its substrates suggesting that it uses different amino acids to interact with different RNAs [36,37]. Together, these studies demonstrate that each of the dsRBDs recognize specific elements in the RNA substrate. 

## 5. The Catalytic Deaminase Domain

The structure of the deaminase domain of hADAR2 has been solved by X-ray crystallography [41]. This C-terminal 45 kDa domain comprises of 400 residues which form a globular structure similar to the deaminase domain found in other CDA family members [42,43,44,45]. This core deaminase motif consists of two α-helices (α2 and α5) and four β-strands (β1, β2, β5, and β8) [41]. Additionally, the active site of this deaminase domain contains one zinc ion and one molecule of inositol hexakisphosphate (IP_6_) (Figure 2b). 

Hydrolytic deamination catalyzed by ADARs uses a nucleophile comprised of a zinc atom (Zn^2+^) and a water molecule (H_2_0). The crystal structure of ADAR2 revealed that the Zn^2+^ atom is coordinated by two cysteines (C451 and C516) and a histidine (H394) [41]. Mutagenic analysis of the predicted Zn^2+^ coordinating residues in ADAR1; His 910, Cys 966, and Cys1036 resulted in complete loss of ADAR1 activity demonstrating the essential role of Zn^2+^ in the catalysis [40]. A highly conserved glutamate residue (E) participates in the proton transfer cascade during deamination. In ADAR2 the glutamate residue (E396) is the acceptor of a proton from the nucleophilic water molecule bound by the zinc atom. This produces a highly-reactive negatively-charged zinc-hydroxide that reacts with carbon 6 (C6) of the adenosine occupying the active site. The removal of a proton from the zinc-hydroxide ion results in the displacement of ammonia (NH_3_) during the reaction. Replacing this glutamate residue with an alanine results in a loss of catalytic activity of ADAR2 [40].

A unique feature identified in the crystal structure of ADAR2 is the presence of an IP_6_ molecule in the core site of the deaminase domain [41]. In the cell, the cleavage of phosphatidylinositol biphosphate by phospholipase C (PLC) generates IP_3_ which is subsequently phosphorylated to produce IP_6_ [46]. In ADAR2, the IP_6_ molecule is located in a basic cavity where it is tightly associated to conserved residues via hydrogen bonds and it is thought to contribute to the folding of the protein [41]. Macbeth and colleagues demonstrate that IP_6_ is an essential ADAR2 cofactor as *S. cerevisae* that are unable to synthesize IP_6_ cannot express catalytically-active ADAR2 [41]. Another study also demonstrated the importance of IP_6_ for ADAR2 activity. Schmauss and colleagues were investigating the regulation of the serotonin receptor (5-HT_2C_) mRNA and observed that an increase in serotonin (5-HT) levels resulted in increased editing of 5-HT_2C_ pre-mRNA [47]. High levels of 5-HT led to the induction of PLC and the subsequent production of IP_6_ which could contribute to the production of ADAR2 which edits the 5-HT_2C_ pre-mRNA. Editing of the 5-HT_2C_ pre-mRNA decreases the production of 5-HT implicating that IP_6_ is a rate limiting factor in this negative feedback regulatory loop [47].

As the edited base lies within the deep and narrow major groove of the A-form RNA helix, it was proposed that the ADARs use a base-flipping mechanism to facilitate the deaminase domain access to the adenine base [48]. The mechanism of recognition of the edited base by ADAR1 and ADAR2 is very similar however ADAR1 is more dependent on the presence of N7 in the edited base [49]. Structural studies of the ADAR2 active site revealed that the architecture of the zinc ion and its coordinating residues would require a conformational change in the RNA before deamination. Stephens and colleagues used a fluorescent RNA probe that is quenched when the RNA helical duplex is naturally stacked and observed a significant increase in fluorescence emission from an RNA substrate bound at the active site of ADAR2 indicating that ADAR2 causes a conformational change at the editing site [50,51]. Another study used molecular dynamics simulations to investigate base-flipping at two sites within the *Gria2* transcript, at an edited and a non-edited site [52]. They found that the adenosine base at the editing site is more flexible and is inclined to move out into the minor groove. Beal and colleagues who were investigating the protein-RNA conformational changes that occur during deamination, found that nucleotides near the editing site on the opposite strand of the RNA become hypersensitive to hydrolytic cleavage after binding by the dsRBD of ADAR2 [53], indicating that RNA binding by ADAR changes the conformation of the RNA. In the same study, a tryptophan fluorescent probe was used to demonstrate conformational changes in the deaminase domain of ADAR2 in the presence of an RNA substrate.

Despite being isolated and characterized over 20 years ago, there is currently no structural information for a full-length ADAR containing both the dsRBDs and the deaminase domains. Nor is there any co-crystal of an ADAR protein with its dsRNA substrate. This is due to the modular nature of these enzymes which is a source of flexibility that makes *in vitro* studies and crystallization experiments more challenging. Another open question in the field is the role of dimerization of ADARs in the function of this family of enzymes. The ADAR proteins were shown to form dimers *in vitro* and *in vivo* [54,55,56]. However, it is still controversial whether the dimerization is essential for the activity [57] and whether it occurs in a RNA-dependent [54,55] or independent manner [58].

## 6. The Two Types of A-to-I RNA Editing

A-to-I editing by ADARs can be classified into two types: site-specific and promiscuous editing. Site-specific editing occurs within short, imperfectly-paired regions of dsRNA that can result in recoding of open reading frames to generate proteins with altered functions. High levels of site-specific RNA editing are found in transcripts that are expressed in the CNS. Since inosines prefer to base-pair with cytosine (C), a guanosine residue is inserted when cDNA is generated. Inosine is read as if it were guanosine by the translational machinery thus recoding the mRNA.

The second type of editing occurs within long dsRNA structures (>100 bp) that are deaminated at multiple sites. It has been demonstrated that up to 50% of the adenosine residues within these long dsRNAs can be deaminated so therefore this type of editing is referred to as hyper or promiscuous editing [48,59]. The deamination of an adenosine within an A-U base pair duplex produces the less stable I-U wobble pair, while deamination within A-C mismatches produces the more stable I-C pairs. These editing events are found within RNA duplex structures formed by inverted Alus and other repetitive elements within the non-coding regions of transcripts such as introns and 3ʹUTR regions as well as within transcribed intragenic regions [60,61,62,63,64,65]. The presence of A-to-I modifications in transcripts encoding Alu elements and other hyperedited transcripts regulate the structure and stability of the dsRNAs and inosine-containing dsRNAs have been shown to play an essential role in regulating the innate immune response [66,67]. This type of editing will be discussed later.

## 7. RNA Editing Resulting in Recoding

When compared to mammals invertebrates have a significantly higher level of editing that results in recoding (for review, see [68]). In octopus, editing of transcripts encoding a potassium channel has been shown to vary depending on the location of the species, being highly edited in cold waters, whereas tropical water species are mostly unedited [69]. This elegant experiment suggests that RNA editing can respond to environmental queues.

The transcript encoding one of the α-amino-3-hydroxy-5-methylisoxasole-4propionate (AMPA) receptor subunit is the best studied mammalian site-specific editing event [70,71]. The AMPA receptor is a glutamate-gated ion channel that mediate fast synaptic transmission at excitatory synapses which are important for neuronal development, long term potentiation and synaptic plasticity. AMPA receptors are activated by L-glutamine which triggers an influx of calcium (Ca^2+^) into the neuron. The Ca^2+^ permeability of the AMAP receptors is dependent on the tetrameric composition of the subunit of the glutamate receptor [72]. AMPA receptors containing the glutamate receptor 2 (GRIA2) subunit exhibit lower Ca^2+^ permeability compared to those without it and this is due to the presence of single amino acid residue, arginine (R) in the pore of the channel [73]. When comparing cDNA and genomic sequences encoding the GRIA2 subunit, it was found that the R codon (CGG) in the cDNA was a glutamine (Q) codon (CAG) in the genomic sequence [70]. ADAR2 is the enzyme that catalyzes this critical site specific editing event at this Q/R site [74]. This editing event also regulates the heterotetrameric assembly of the AMPA receptors by retaining the GRIA2 subunits within the endoplasmic reticulum therefore increasing the time required for it to be transported to the synaptic surface [75,76] Editing at this Q/R site is highly efficient >99% in the CNS and it dictates the Ca^2+^ permeability of the AMPA receptor. It is therefore is a key determinant in neuronal Ca^2+^ homeostasis, as the edited isoform, GRIA2 (R), is impermeable to Ca^2+^.

The early onset epileptic seizures and premature death of both *Adar2* null mice and mice heterozygous for an uneditable *GRIA2* allele demonstrated the physiological importance of this editing event [77,78]. The *Adar2* null phenotype is rescued by generating a transgenic mouse containing the edited *GRIA2* allele [71]. In addition to the Q/R site, the *GRIA2* transcript is also edited at the R/G site [79]. The transcript encoding the GRIK2 subunit of the high affinity kainite (KA) receptor is edited at the Q/R site in transmembrane segment 1 (TM1) and also at the I/V and Y/C sites in TM2 [80]. Editing at these positions affects the Ca^2+^ permeability of the KA receptor channel. In contrast to the AMPA receptors, editing at the Q/R site of the *GRIK2* transcript increases the Ca^2+^ permeability of the KA receptor [80]. Burns and colleagues demonstrated that transcripts encoding the G-protein-coupled 5-HT_2C_ undergo editing at sites within the second intracellular loop which results in 3 amino acid changes of isoleucine to valine (I/V), asparagine to serine (N/S) and isoleucine to valine (I/V) [81]. RNA editing at these sites generates several 5-HT_2C_ isoforms which are expressed at various levels in different brain regions [81]. The fully edited 5-HT_2C_ isoform has a reduced affinity for the G-protein PLC which inhibits the downstream phosphatidylinositol signal pathway [81]. Therefore, RNA editing regulates the efficacy of receptor-G-protein coupling. The *Kv1.1* transcript encoded by the voltage gated potassium channel gene is edited at a single site and results in an isoleucine-to-valine (I/V) recoding event [82]. The I/V site is located in a highly conserved region of the channel pore that is involved in modulating potassium ion flow and RNA editing at this site results in the fast inactivation of the Kv1.1 channel [82]. The *Gabra-3* transcript encoding the α3 subunit of the GABAA receptor undergoes site-specific editing, resulting in an isoleucine to methionine (I/M) amino acid change [83]. RNA editing at this site increases during development and editing levels are inversely related to expression of the α3 subunit containing GABAA receptor [84].

Overall, the highest levels of site-specific editing events in mammals occur within transcripts expressed in the CNS, affecting the functional properties of the proteins. However, the mechanism through which ADARs is able to identify a specific adenosine within these transcripts is not yet understood.

## 8. RNA Editing in Alu Repeated Sequences

The continuing development of high throughput sequencing methods has led to major improvements in the detection levels of genome-wide editing events. To date it has been estimated that there are over one hundred million editing sites in the human genome [85], and the whole transcriptome analysis has revealed that A-to-G changes account for greater than 90% of the total mismatches identified in humans [64,86], editing that result in recoding account are rare amongst the total number of editing sites identified in the human transcriptome. However the level of editing at site-specific sites is high whereas editing of repeat sequences is general low but widespread.

*Alu* elements are primate specific non autonomous short interspersed nuclear elements (SINEs) which make up approximately 10% of the human genome [87]. The *Alu* element is approximately 300 bp comprising of an A-rich region. *Alu* repeats are generally clustered in gene rich regions, within UTRs and introns [88]. Due to their repetitive nature *Alus* are capable of forming long intramolecular duplex structures through complementary pairing between antisense repeats found within a single pre-mRNA. Often two Alu elements are present, adjacent to each other in reverse orientation in the genome which are ideal substrates for ADARs. SINE families in mice are not as evolutionary conserved as the primate SINE *Alu* elements so they do not base pair as well. Therefore the editing levels of SINE transcripts in mice are lower [65].

The functional consequences of editing *Alu* transcripts in primates remain elusive. The proportion of A-to-I editing events that destabilize an A-U pair to an I-U as opposed to stabilizing an A-C pair to an I-C is 4:1 respectively [62]. Therefore A-to-I modifications in *Alu* repeats, regulates the structure and stability of the dsRNA formed by *Alu* elements.

*Alus* are mobile elements that retrotranspose through RNA intermediates back into the human genome which can result in the disruption of genes [89]. It is possible that the clustering of RNA editing sites within individual transcripts could prevent this transposition of *Alus* [62]. This may also be relevant to retroviruses as A-to-I editing by ADARs may protect the host genome from infection by preventing their integration.

Bazak and colleagues found that of the 23,357 human RefSeq genes approximately 14,715 harboured editable *Alus* and 2896 contained *Alus* in exonic regions [85]. Therefore, it is not surprising that A-to-I editing of *Alus* elements leads to transcript variants in *Alu*-harboring gene [85,90]. Based on the number of edited *Alus* elements and as there can be up to 11.5 inosines per *Alu* transcript, A-to-I editing of *Alu* elements undoubtedly is a major contributor to transcritpome diversity [85].

## 9. RNA Editing in miRNA

The miRNA biogenesis pathway is targeted by ADARs at various stages and this has an impact on miRNA processing and miRNA-mediated silencing (For review [91]). A-to-I editing can affect both Drosha and Dicer mediated cleavage. For example pri-miR-142, expressed in hematopoietic cells is edited by ADAR1p110 and ADAR2 [92]. Yang and colleagues demonstrated that edited pri-miR-142 could not be processed by Drosha and instead is degraded by Tudor-SN [92]. The pri-miR-151 is also subject to RNA editing by both isoforms of ADAR1. However, editing of pri-miR-151 inhibits cleavage by Dicer and accumulation of edited pre-miR-151 RNAs therefore suppressing the expression of mature miR-151 [93].

A-to-I editing by ADARs also affects miRNA-induced silencing complex (miRISC) assembly, antagonizing miRNA-mediated gene silencing. For instance, the Epstein Barr virus encoded pri-miRBART6 is edited by ADAR1 and this reduces the loading efficiency of miR-BART6 into miRISC which in turn reduces the silencing of its target mRNA [94].

The target specificity of mature miRNAs can also be regulated by A-to-I editing. The miR-376 is edited by ADAR2 within its “seed” sequence (+4 site), an essential region for RNA duplex formation with the complementary target mRNA [95]. Kawahara and colleagues demonstrated that A-to-I editing of pri-miR-376 results in the redirection of its silencing effects to a different set of target mRNAs. Additionally, ADARs can regulate miRNA processing in an editing-independent manner as the catalytically inactive ADAR2 mutant inhibits Drosha processing of pri-miR-376a2 through RNA-binding [96]. Ota and colleagues revealed that ADAR1 forms a heterodimer complex with Dicer to promote both the cleavage of pre-miRNA by Dicer and the 52 subsequent miRISC mediated gene-silencing events [97]. However, this complex has no A-to-I editing activity and, therefore, is another RNA editing-independent role of ADAR1 in the RNAi pathway. Together these results demonstrate that the RNA editing and RNAi pathways are antagonistic as they compete for similar dsRNA substrates.

## 10. ADARs and the Innate Immune Response

**Figure 3:**
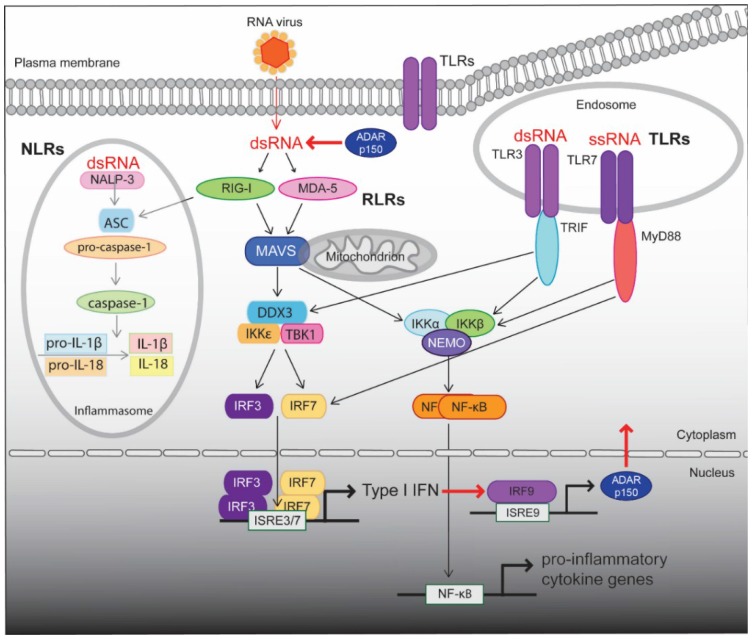
Intracellular detection of viral RNA by PRRs and activation of the innate immune response. The three classes of PRRs responsible for detection of viral RNA are the TLRs, the RLRs, and the NLRs. In the endosome viral ssRNA is detected by TLR7 whereas viral dsRNA is recognized by TLR3. TLR7 recruits the signaling adaptor MyD88 whereas TLR3 associates TRIF. MyD88 elicits the production of type I IFNs through the direct activation of IRF7. TRIF activates the TRAF family member-associated TANK and IKKε both of which undergo phosphorylation to activate both IRF3 and IRF7. Both IRF3 and IRF7 form homodimers which translocate to the nucleus leading to the induction of type I IFN. At the same time, both TRIF and MyD88 activate IKK. The IKK complex triggers the release and translocation of NF-κB to the nucleus leading to the production of pro-inflammatory cytokines. In the cytoplasm, short dsRNA with 5'triphospahte ends is detected by RIG-I and long dsRNA is sensed by MDA5. Activation of RIG-I and MDA5 results in the recruitment of MAVS via CARD domain interactions. MAVS dependent signaling causes the translocation of the transcription factors IRF3 and NF-κB for the induction of type I IFN and pro-inflammatory cytokines respectively. The inflammasome is activated by dsRNA via NALP3-dependent signaling pathway. NALP3 together with ASC recruit pro-caspase-1 which in turn undergoes auto-cleavage to produce caspase-1. Activation of caspase-1 is required for the cleavage of pro-IL-1β and pro-IL-18, and subsequent secretion of the proinflammatory cytokines IL-1β and IL-18, respectively. ADAR1p150 edits dsRNA within the cytoplasm and is induced by IFN.

The innateimmune system is the first line response to a pathogenic infection and mounts the rapid activation of the initial defense response (For review [98]). The early detection of threat by the cell involves the recognition of pathogen associated molecular patterns (PAMPs), which are unique to the invading pathogen. These PAMPs are “sensed” as foreign by the host’s specific pattern recognition receptors (PRRs). Upon identification of a threat, these cellular detectors recruit specific adaptors which activate various signaling pathways to propagate defense signals downstream, ultimately triggering the appropriate immune responses in the form of type I IFNs, pro-inflammatory cytokines and chemokines (Figure 3). PRRs are classified by their localization within the cell and by the specific type of ligands. Three classes of PRRs that detect viral RNA have been discovered: the Toll like receptors (TLRs), the retinoic acid-inducible gene-I (RIG-I) like receptors (RLRs), and the nucleotide-binding oligomerization domain (NOD)-like receptors (NLRs). The RLRs, retinoic acid inducible gene I (RIG-I) and melanoma differentiation-associated gene 5 (MDA5), are located in the cytoplasm. They are both IFN-inducible DExD/H box RNA helicases with an RNA binding helicase domain at the C-terminus and two caspase activation and recruitment domains (CARDs) at the N-terinmus [99,100]. Activation of RIG-I and MDA5 results in the recruitment of the mitochondrial antiviral signaling protein (MAVS) via CARD domain interactions. This leads to the translocation of the transcription factors IRF3 and NF-κB and the induction of type I IFN and pro-inflammatory cytokines respectively. IRF3 is essential for the transcription of type I IFNs in response to viral infection [101].

A number of studies have demonstrated the involvement of the ADAR proteins and A-to-I editing during viral infection [102,103,104,105]. As the majority of viruses replicate within the cytoplasm, they are edited by the IFN-inducible isoform of ADAR1, whereas viruses that replicate in the nucleus can be edited by either ADAR1p110 or ADAR2 [106]. Depending on the type of virus, the biological consequences of A-to-I editing of viral RNA can be either antiviral or proviral [106].

Viral dsRNA can be generated from viruses as a result of their genome (dsRNA viruses); hairpin secondary structures (viral mRNA), viral transcription (DNA viruses), and viral replication (ssRNA viruses). In the last decade many cellular dsRNAs have been found which are derive from different processes, such as bidirectional transcription, as well as from repetitive sequences that, when transcribed, can create a source of dsRNA. Therefore, the innate immune system must recognize some molecular features to distinguish endogenous dsRNA from viral dsRNA.

Investigations into the biological role of ADAR1 have found that it is a negative regulator of the type I IFN response. The *Adar1* null mouse have defects in hematopoiesis and liver disintegration as well as elevated levels of type I IFN and widespread apoptosis [107,108,109]. Mouse embryonic fibroblasts (MEFs) deficient in ADAR1p150 are highly susceptible to infection and have enhanced viral induced cytotoxicity effects [105], implicating ADAR1 as being involved in type I IFN response.

The *Adar1* null mice are dead by stage E12.5 and for over 10 years investigators endeavored to elucidate what caused this early lethality [66]. They presumed that these mutant mice lacked an essential editing event that would be analogous to ADAR2 editing the Q/R site of *GRIA2*. A major breakthrough was achieved by taking a genetic approach and generating a double mutant with *Adar1* [66]. When *Adar1* heterozygous mice were crossed with mice that were homozygous for *Mavs*, the embryos that were double-homozygous survived to birth. The *Adar1* mutant embryos had a heightened IFN response whereas the double mutants had a response that was similar to their *Mavs* parents. This places *Adar1* upstream of RIG-I and MDA5 in the innate immune pathway. As MEFs from *Adar1* were not viable, MEFs were generated from embryos that were *Adar1:p53* double mutants. When these MEFs were stressed they had a heightened immune response however the addition of inosine containing dsRNA alleviated the heightened immune response [66,67]. The explanation for these results is that the cell uses inosine to help discriminate between self and non-self dsRNA. If inosine is present in dsRNA it binds to -RLRs and prevents activation of the innate immune response. However, if no inosine is present, then the cell recognizes the dsRNA as being of viral origin and mounts a type 1 IFN response.

## 11. ADARs in Human Disease

Aicardi-Goutiéres syndrome (AGS) is an autoimmune disorder where patients develop severe inflammatory encephalopathy due to chronic activation of the type I IFN [110] (Table 1). AGS patients present with symptoms that are similar to an acquired infection. However, there is no infection and mutations in genes involved in the regulation of nucleic acid metabolism have been identified in patients with this disorder [111]. Therefore, the proposed mechanism of disease pathogenesis is thought to be the aberrant activation of the innate immune response caused by the intracellular accumulation of nucleic acids [112]. Mutations in ADAR1 have been identified in a subset of patients with AGS [113] and these mutations have been shown to reduce the levels of RNA editing. There is a greater reduction in editing when these mutations occur in the IFN-inducible ADAR1p150 isoform compared to the constitutively expressed ADAR1p110 isoform [66]. This explains the observation that, in some patients with an *ADAR1* mutation, AGS occurs after a viral infection when the ADAR1p150 isoform would be expressed [114]. Mutation in other genes also cause AGS with elevated IFN levels and these are; *TREX1*, *SAMHD1, RNASEH2A, RNASEH2B, RNASEH2C*, and *IFIH1*(MDA5) (for review [115]).

**Table 1:** List of diseases associated with human ADARs.

Disease	*Edited transcripts*	*ADAR*	*Reference*
Prostate cancer	*PRUNE2/PCA3*	ADAR1/ADAR2	[127]
Hepatocellular carcinoma (HCC)	*AZIN1*	ADAR1	[128]
	*FLNB*	ADAR1	[130]
	*COPA*	ADAR2	[130]
	*pre-miR-214*	ADAR2	[131]
Chronic myeloid leukemia (CML)	*PU.1 (Spi1)*	ADAR1	[132]
Glioblastoma (GBM)	*CDC14B*	ADAR2	[135]
	*pri-miR-221/222*	ADAR2	[139]
	*Pri-miR-21*	ADAR2	[139]
	*miR-376a-5p*	ADAR2	[140]
	*GRIA2*	ADAR2	[141]
Aicardi-Goutiéres Syndrome (AGS)	?	ADAR1	[113]
Dyschromatosis Symmetrica Hereditaria (DSH)	?	ADAR1	[116]
Amyotrophic lateral Sclerosis (ALS)	*GRIA2*	ADAR2	[122]

## 12. Dyschromatosis Symmetrica Hereditaria (DSH)

DSH is an autosomal dominant disorder characterized by hypopigmented and hyperpigmented macules localized on the back of the hands and feet in addition to small freckle-like hyperpigmented macules on the face [116,117]. The skin lesions usually appear during infancy and generally stop spreading before adolescence, lasting the patient’s lifetime [118].

Single-strand conformational polymorphism analysis (SSCP) of the candidate genes within this locus revealed that each of the patients were heterozygous for mutations in the gene encoding ADAR1 [119]. After this first report showing that *ADAR1* was the causative gene of DSH, more than 130 mutations in the *ADAR1* gene have been identified in DSH patients. The mutations include: missense, non-sense, frameshift insertions, frameshift deletions, and splice-site. They are not confined to certain hotspots within *ADAR1*; rather, they are spread along the gene. Patients with DSH also have an increased expression in IFN transcripts, however, the level is not as high as observed in AGS patients [113].

## 13. Amyotrophic Lateral Sclerosis (ALS)

Amyotrophic lateral sclerosis (ALS) is a progressive neurodegenerative disorder that is characterized by the selective death of motor neurons. ALS is a fatal disease with a survival rate of less than five years subsequent to diagnosis and patients usually die as a result of respiratory failure. Mutations in the superoxide dismutase gene (SOD1) have been associated with approximately 20% of the familial cases of ALS [120]. However, less than 10% of ALS cases are inherited and the majority of patients develop the disease sporadically. Sporadic ALS is characterized by the presence of TAR-DNA binding protein (TDP-43)-containing cytoplasmic inclusions in the spinal motor neurons however its pathogenesis remains unknown [121].

Kawahara and colleagues analyzed the editing levels at the *GRIA2* Q/R site in individual motor neurons of patients with sporadic ALS, and they observed a significant decrease in the editing levels of the Q/R site in *GRIA2* transcript when compared to controls [122]. As ADAR2 is the enzyme responsible for editing the Q/R sites in *GRIA2* transcripts, these results suggested that defective ADAR2 activity could be the causal mechanism of the editing deficiency observed in patients with ALS. Later, a conditional transgenic mouse model was generated in which there was loss of *Adar2* in motor neurons (AR2 mouse model) [123]. These mice exhibited defects in motor function due to the death of *Adar2*-deficient motor neurons in the spinal cord. The specific expression of ADAR2 in the AR2 mice successfully prevented motor neuron death and rescued motor neuron function [124].

## 14. A-to-I Editing in Cancer

Consistent with the essential roles played by ADARs *in vivo*, it is likely that uncontrolled nucleotide changes mediated by these enzymes could predispose cells to developmental defects and potential oncogenic outcomes. Using both bioinformatics and experimental approaches, it has been found that the A-to-I editing pattern is significantly modified in cancers compared to normal tissues [125], with some cancers having over-editing and others down-editing events [126]. Here, we discuss some studies of the emerging roles of A-I editing in cancer biology (Table 1).

## 15. Prostate Cancer

Prostate cancer antigen 3 (PCA3) is the most specific prostate cancer biomarker and urinary PCA3 testing has the potential to significantly decrease the number of unnecessary prostate biopsies. However PCA3 function remains unknown. PCA3 is an antisense intronic long noncoding (lnc)RNA with the potential to base-pair with the sense PRUNE2 transcript. PCA3 expression controls PRUNE2 levels via a unique regulatory mechanism involving the formation of a PRUNE2/PCA3 dsRNA that is edited by ADARs [127]. PRUNE2 and PCA3 elicited opposite effects on tumor growth in immunodeficient tumor-bearing mice. Co-regulation of PRUNE2/PCA3 RNA editing was also confirmed in human prostate cancer specimens. These results indicated that PCA3 may act as a dominant-negative oncogene, whereas PRUNE2 is a possible tumor suppressor gene in human prostate cancer [127].

## 16. Hepatocellular Carcinoma (HCC)

In hepatocellular carcinoma (HCC) increased editing of the transcript encoding antizyme inhibitor 1 (*AZIN1*) by ADAR1 results in increased cell proliferation [128,129]. ADAR2 may also play a role in liver tumours as patients with ADAR1 overexpression and down-regulation of ADAR2 have a further increase risk of liver cirrhosis and post-operative recurrence with poor prognoses [130]. Hyper-editing of *FLNB* and hypo-editing of *COPA* transcripts are associated with HCC pathogenesis in a specific HCC subset [130]. A study investigated whether ADAR2 may affect miRNA expression in HCC focused on a subset of HCC with elevated expression of ADAR2 and found that that ADAR2-mediated editing events within pre-miR-214 resulted in decreased levels of mature miR-214 [131]. In this subset of cancer tissues, this caused increased protein levels of the novel target of miR-214 which was identified as *Rab15*.

## 17. Chronic Myeloid Leukemia (CML)

Chronic myeloid leukemia (CML), also known as chronic granulocytic leukemia (CGL), is a myeloproliferative disease associated with a characteristic chromosomal translocation; the Philadelphia chromosome. This translocation leads to a fused BCR-ABL protein being able to activate cell proliferation through modulation of the cell cycle. Jamieson and colleagues demonstrated that during disease progression, there is increased expression of the IFN-γ pathway and among the elevated IFN-genes is ADAR1 p150 [132]. They found that over-expression of ADAR1 p150 directly correlates with the up-regulation of the myeloid transcription factor PU.1. However, ADAR1 knockdown in CML progenitors, impaired *in vivo* the self-renewal capacity of the cells. The transcript encoding PU.1 (*Spi1*) has multiple editing sites; however, the authors did not investigate the mechanism of how the editing enzymes modulate the expression of PU.1. Another study of CML with an *Adar1* mutant mouse model found that ADAR1 deletion reversed leukocytosis and preferentially-depleted primitive Lin-Sca+Kit+ (LSK) leukemic cells [133], which would imply that ADAR1 should become a new molecular target for CML-directed therapeutics. However, considering the critical role of ADAR1 in the innate immune response this approach should proceed with caution [66].

## 18. Glioblastoma

Several studies have shown a decrease in ADAR2 editing activity in glioblastoma [125,134,135,136,137], whereas the rescue of ADAR2 in glioblastoma cells inhibited cell proliferation and migration *in vitro*, and more importantly *in vivo*, with a 100% survival in a mouse model [134,135]. Editing by ADAR2 inhibits the growth of glioblastoma tumors, indicating the importance of editing of target transcripts for glioblastoma regression and possible future therapeutic intervention. The phosphatase CDC14B, which acts upstream of the Skp2/p21/p27 pathway, is a critical ADAR2 target involved in glioblastoma growth [135]. The decreased ADAR2 activity in glioblastoma causes the loss of the edited miRNA pool and the unbalanced expression of several miRNAs with the subsequent up-regulation of several onco-miRNAs [138,139]. In particular, ADAR2-editing events within precursors of onco-miRs (pri-miR-221/222 and pri-miR-21) are able to significantly decrease the level of mature miRNAs, leading to a significant decrease in cell proliferation and migration [138,139] (Gallo unpublished data).

Decrease of ADAR2 activity in glioblastoma can also affects miR-376a-5p activity. High-grade gliomas accumulate the unedited form of miR-376a-5p, which promotes cell migration and invasion while edited miR-376a-5p suppresses these features [140]. A search for specific targets important in glioma cell migration found that down-regulation of *RAP2A* **(**Ras-related protein Rap-2A) and up-regulation of *AMFR* (autocrine motility factor receptor) expression promote *in vitro* migration and invasion of glioma cells [140]. As additional target for glioma progression was identified in the *GRIA2* transcript which is edited by ADAR2 [71], where less editing at the Q/R site enhances glioblastoma invasion through activation of the Akt pathway [141]. Overall, these data indicate the essential role of ADAR2 editing in glioblastoma, acting on multiple targets (*CDC14B,* pri-miR-221/222, pri-miR-21, miR-376a-5p, *GRIA2*) that together contribute to varying extents to cancer progression.

## 19. Conclusions

In the last 25 years a lot has been learned about the ADAR family of proteins; however, many intriguing questions remain. Despite considerable effort, there is no co-crystal structure of an ADAR enzyme bound to dsRNA. How do the enzymes recognize the specific adenosine to edit in transcripts? Are there additional co-factors and how do they influence RNA editing?

Perhaps the most intriguing question is; are the ADAR proteins heroes or villains? They appear to be essential for recoding some neuronal transcripts and mounting an appropriate innate immune response, but they also play a significant role in the progression of different types of cancer. Perhaps they play a role in both, as in the words of Tennessee Williams, *“If I got rid of my demons I’d lose my angels”*.
